# Infant Mortality, Its Prime Cause and Prevention*Read before Atlanta Society of Medicine, May 4, 1897.

**Published:** 1897-07

**Authors:** E. Van Goidtsnoven

**Affiliations:** Atlanta, Ga.


### INFANT MORTALITY, ITS PRIME CAUSE AND PREVENTION.*

BY E. VAN GOIDTSNOVEN, A.M., M.D.,

ATLANTA, GA.

Vital statistics prove that one-third of all deaths are of infants under one year; that one-third of those who fall sick die during the first year; that of the remaining two-thirds few escape a severe sickness, and about ten per cent. more die before reaching two years; that one child in three dies before the end of the fifth year. These statistics are indisputable, taken from the official returns of the boards of health of the leading cities of this country and form the basis of computations in life insurance. In juvenile insurance no child under one year of age is admitted. The risk is too great. Forty per cent. of the deaths that occur in the first year are due to diseases of the digestive organs, and twenty per cent. to diseases of the respiratory organs. In the second year the respiratory organs are more exposed to danger. So that in the first year the stomach and intestines, and in the second, the bronchi and the lungs, are the sources of the high death-rate.

Two most interesting facts are elicited from a study of mortality

*Read before Atlanta Society of Medicine, May 4, 1897.

tables. One is the enormous increase of mortality during the heated season; the other the comparatively low death-rate of the much-dreaded second summer. For this reason I shall limit the scope of this paper to the consideration of the pathological conditions of the first year of infantile life.

Hitherto excessive heat, defective ventilation and teething have been assigned by the profession and accepted by the laity as the chief lethiferous factors. But it is now a conceded fact that this destruction of infant life in its earliest period is due almost solely to either faulty or excessive alimentation.

It is a just reproach to our modern civilization, that with the wonderful progress it exhibits in so many things tending to promote material well-being, there should be so little popular understanding and appreciation of the line of conduct that should guide every home in averting so much grief, desolation and misery, consequent upon the death of a child.

Experience is a great adjuvant when reinforced by scientific attainments. Otherwise, it leads to quackery, empiricism and death; withers everything it touches, and its influence, like that of the Parcæ, dots our cemeteries with countless graves; it teaches the impudent and baneful theory that disease is an entity, an evil spirit that can be driven out by drugs; that each case of a given disease is the same; that what cures one will cure another; that a cure for disease can be warranted; that animate nature should be driven by scales, and weights, and wooden yardsticks; that increasing weight and rotundity are synonymous with increased health and strength; that a lean baby should be fat in order to be healthy, and fed as though it were to compete for a prize at the next fair or exposition.

Under these circumstances it is little to be wondered at that gastric and intestinal diseases, followed by general wastings, are so alarmingly prevalent. Compare the condition of infantile life with that of the young of the brute creation, and you will admit that the reasoning faculties of man are sometimes defeated by the natural instincts of animals, for the latter not only feed their progeny on natural food from the beginning, and without any excess, but they *do not know enough to kill them.* The moral to be drawn is that no theory, no experience, can be in opposition to nature's laws, to scientific truths, and in this instance to physiological, hygienic, and dietetic dictates, so long, at least, as we have a rate of mortality which cannot be contemplated without a shudder and is due to causes mostly preventable.

An infant may be fed in one of three ways: 1. From the mother's breast. 2. From the breast of a foster mother or wet nurse. 3. From the bottle by the method known as the artificial or hand feeding. Lactation, being a physiological process, is not a drain upon the systemic strength so long as the functions of nutrition are actively performed. It follows that, as a rule, a woman who can bear a child can nourish it. Mothers do not seem to understand that the nature who provides organs for bearing children also provides organs for nursing children, and intends that these organs should be for use rather than for ornament. They do not realize that the products of modern science at best are but poor substitutes for the products of nature. "Heaven sends the food," says a humorist, "but the devil sends the cook." Aptly might it be said in this instance that heaven supplies the mammæ, but the devil suggests the artificial food. The ancients have handed down to us a maxim which, for force and terseness, surpasses anything I have ever read respecting this maternal duty: "*Quæ lactat mater magis quam quæ genuit*," "She who gives suck is a truer mother than she who gives birth."

It cannot be disputed that breast-milk is the food par excellence and naturally presents, or rather should invariably present, the physiological type of nourishment required by the infant. Mother's milk is the normal, physiological diet of infants previous to and during the greater part of the period of dentition both in the state of health and of disease. In nature's laboratory, where this diet is prepared, there can be no such thing as sophistication, adulteration and tempering. Attempts have been made and are continually being made, with more or less success, to produce and furnish a substitute for the physiological mammary secretion. But too often disordered disgestion, marasmus and starvation result.

But many a morbid condition may affect the mother's milk, and therefore not agree with the baby. The milk may be unreliable either in quantity or in quality. With respect to quantity, it may be stated that in many cases absolute deficiency of milk is quite compatible with defective development of the mammary glands. Anemia is a frequent cause of deficiency. Irritation of the nervous system, depressing emotions, violent excitement, dissipation, anxiety, prevent an equal flow of blood, and therefore of milk.

The quality of milk is of a paramount importance. Consumption, scrofula, syphilis, anemia, chronic eruptions, prolonged lactation, medicinal substances, pregnancy, menstruation, hereditary transmissions, produce vital changes in the chemical composition of the milk, which pervert the nutrition of the nursling and are evidently to be dreaded, for illness in a nursing woman means poor milk and a sickly baby.

Shall a wet nurse be secured? A reliable wet nurse is a rare bird, especially if it be of a dark plumage. Seventy-five per cent. of the negro race is either tuberculous, scrofulous, or syphilitic! This may be a startling revelation to some, but it is nevertheless true. Therefore, beware! One should by all means have a wet nurse examined by a physician before engaging her services, and the best recommendations respecting her character and moral conduct should be received with a grain of salt. It should be borne in mind that the milk of an excitable woman is dangerous to a child. Lastly, the date of the nurse's confinement is an important consideration in estimating the value of her milk.

Let us suppose then that for some excellent reasons mother's milk is not obtainable and the wet nurse question has been decided adversely. Upon what and how shall the infant be fed? As stated above, mother's milk is undoubtedly the proper food for the new infant. Any substitute should therefore be equivalent in composition to mother's milk, in order to perfectly fulfill every requisite that nature demands as a food for infants.

The logical consequence is that we should be familiar 1st, with all the component parts of human milk; 2d, with their relative proportions; 3d, with their chemical and physiological properties. The following table, furnished by König, shows the component parts of human milk:

| | | |
|---|---|---|
| Water | | 87.09 |
| Sugar | | 6.04 |
| Albuminoids | Casein | 0.63 |
| | Albumen | 1.31 |
| Fats | | 3.90 |
| Salts | | 0.59 |

Reaction alkaline.

Thus we see that milk is composed of five different classes of material which are essential to nutrition. Eighty-seven parts in a hundred of baby's food is water. But when the chemist tells us that seventy per cent. of the human body weight is water, its importance is readily recognized. Then we have the nitrogenous group represented by the albuminoids, the muscle forming; and then the fat and sugar or carbohydrates which maintain the animal heat, and the salts for bone and secretions.

In consulting the different authorities on infant feeding, we find some discrepancy in the analysis of human milk, showing that it is not constantly uniform in its composition. It should be borne in mind that many conditions will influence the composition of the milk, varying from a slight loss in the percentage of some of its constituents to a profound chemical change. We should also be impressed with the important fact that gradual changes take place in the constitution of mother's milk, which are adapted to the successive requirements of the growing infant. This marvelous adjustment of human milk to the baby's needs is a fixed law of nature which cannot be disturbed without interfering with the growth and development of the nursling. According to Leeds this variability affects mostly the proportion of the albuminoids, but always leaves that of the carbohydrates unimpaired, by which nature intends to teach us that the rate of nutrition in an infant may safely vary within wide limits, but that of the animal heat of a highly organized and rapidly developing creature must be maintained at all hazards. However, the above table can be relied on as being the average composition of human milk.

It must be admitted that science has of late made rapid strides in its efforts to prepare substitutes for mothers' milk, with more or less degrees of accuracy, and we are indebted to chemistry for various preparations, which in many instances almost reach every requirement of a complete physiological food. The majority of people possess a large measure of common sense, yet may not be sufficiently enlightened to know that chemistry alone is unequal to pronounce on the nutritious possibilities of any artificial food. The public may not be aware that there is such a thing as isomerism; that is to say, that two or more compounds may have the same composition, the same molecular weight, and the same general reaction, yet differ in their physical and chemical properties, owing to the different arrangement and grouping of their atoms. This constitutes a difference of organic form affecting vitality, and is often the sole reason why one substance may be nourishing and the other hurtful. There hardly ever exists a constant relation between a chemical analysis of a compound and its therapeutic or nutritive value. Hence, in the language of Jacobi: "It is not the formula alone which determines the rank of a substance as a nutriment; it is not exclusively the chemistry of an article, but its digestibility which comes into question."

Back of physiological analysis there is a mysterious and vital action that is not revealed even by the microscope. I hold that animal milk forms a harmonious whole which chemistry does not obtain; that there exists between animal milk and the principles it contains a relation which has thus far baffled the best efforts of chemical erudition and skill; that an organic element, a vital principle, holds its habitat therein, and has been either lost sight of or ignored. May we not, then, infer that the nutritive and vitalizing value of animal milk should in a great measure be assigned to that element? Therefore, I believe that natural products, and not artificial ones, should be selected as substitutes for mothers' food; and none come nearer thereto than the milk of mares, asses and cows.

"There are," says Dr. Chapin, "certain requisites for an infant food before it can be recommended for general use. Thus it must be readily procurable under the common and ordinary conditions of life; it must be digestible, nourishing and fairly cheap. The mixing or preparation of such a food must not be complicated. The conditions here mentioned can only be met by employing cow's milk more or less diluted or altered, according to the necessities of the case."

Cow's milk is universally within reach, and on that account preferred by the profession and the laity. But it demands careful selection and intelligent preparation. Any one interested in infant feeding should have a clear conception of the resemblance and difference between cow's milk and human milk.

The composition of cow's milk, as given by König is as follows:

| | | |
|---|---|---|
| Water | | 87.41 |
| Sugar | | 4.92 |
| Albuminoids... | Casein | 3.01 |
| | Albumen | 0.75 |
| Fats | | 3.66 |
| Salts | | 0.70 |
| Reaction acid. | | |

From this table we deduce the following important points: 1. That human milk is richer in sugar and fats than cow's milk. 2. That it is poorer in albuminoids and salts. 3. That it is alkaline. 4. That cow's milk is acid.

In substituting cow's milk for human milk, it is of the utmost importance to modify the former so as to closely resemble the latter. The proportions of water in both human and cow's milk are almost alike. Digestion is solution by hydration, so that the elements acted upon may pass readily through the walls of the alimentary canal, after which they are dehydrated. Sugar is required as an admixture to cow's milk. Jacobi claims it should be cane and not milk-sugar, as the latter increases to an undue extent the amount of lactic acid, and thereby removes alkalies and produces dyspepsia. We know, clinically, that good results are often obtained by adding cream (fat) to the nursing bottle. Fats and sugar constitute the carbohydrates, and contribute animal heat. The latter is readily lost by the infant, and is very essential to its rapid growth and delicate organization. The casein of cow's and woman's milk differs both chemically and physiologically. According to Jacobi there ought to be not more than one per cent. of casein in every infant food. The difference of albuminoids in both cow's and human milk is not one of quantity only. The albuminoids of human milk form in the stomach, when acted on by the gastric juice, a coagulum which is soft and flocculent, and consequently easily digested. Not so with the albuminoids of cow's milk; they coagulate into hard firm masses that require considerable muscular exertion on the part of the stomach to break up and digest them. This fact is an important one in the etiology of infantile gastric and intestinal disorders. To secure in the digestion of cow's milk the preliminary formation of the soft, flaky curds which characterize the mother's milk, it should be attenuated, emulsified, as it were, and blended with oatmeal or barley water, gelatine, or other mucilaginous preparation. Simple dilution will not contribute to this result. Yet it is a necessary and essential adjuvant; and when added to the amount of once or twice its bulk, will so affect the percentage of casein in cow's milk as to bring it to that of the woman, and also will control the precipitation of the curd. Water should be previously filtered. Infant foods ought to be quite fluid. Most infants and children, both well and sick, are supplied with too little water; but this should be done within due proportion, as an excess of water will reduce the amount of carbohydrates. Dilution of milk corrects also the excess of salts in cow's milk, to render it adjustable to infant feeding. The last constituents of milk are the inorganic salts, chloride of sodium and of potassium, phosphate of potassa, iron and lime. But for dilution, the increase of salts in cow's milk would be excessive, and diarrhea would set up in the effort to eliminate the effete matter, just as in starvation. However, Jacobi urges the necessity of adding chloride of sodium (or common table salt) to cow's milk and cereal food. There is found in the blood, and therefore in the milk, of herbivora an abundance of potassium salts, which needs antagonizing by sodium, for the purpose of elimination. Decoctions of barley and oatmeal require addition of table salt. Through the latter the prevalence of potassium is rendered less harmful, osmosis is induced and promoted, and nutrition is everywhere quickened. Another very important point is that chloride of sodium delays and renders difficult the firm curdling of the milk.

Owing to cow's milk being more or less acid, the addition of an alkali is essential. Bicarbonate of soda or lime water will meet this indication. Lime salts counteract all tendency to rickets, and on this account should be given the preference. The degree of dilution necessarily depends upon the composition of the milk, the age and condition of the child.

Age 1 month, 1 part of milk to 3 parts of water.

Age 2 months, 1 part of milk to 2 parts of water.

Age 3 months, 2 parts of milk to 2 parts of water.

Age 6 months, 3 parts of milk to 2 parts of water.

Age nine months, 3 parts of milk to 1 part of water.

Age 12 months, 6 parts of milk to 1 part of water.

Jacobi's food is my favorite, and I never fail to recommend it. Its directions are as follows:

"Boil a teaspoonful of powdered barley (grind it in a coffee mill) and a gill of water with a little salt for fifteen minutes, strain it and mix it with either one-third or one-half or equal part of boiled cow's milk, according to the above table, and a small lump of white sugar. Give it lukewarm through a nursing bottle. Keep bottle and mouthpiece in a bowl of water when not in use. Our choice of the cereals must depend upon the condition of the child. If there be looseness of the bowels, barley, which is deficient in fat, will make the best attenuant. When constipation is present, oatmeal (the same quantity as barley) will serve as a laxative and an attenuant."

The mixed milk of a whole dairy is preferable to the milk of one cow. In giving it the preference, we dilute and diminish dangers which would otherwise attend changes effected by feeding and possible sickness in one cow. Boiled milk is more digestive than raw, especially for children under one year. Boiling is germ destroying. Human milk is free from bacteria. Cow's milk contains numerous bacteria when it reaches the consumer, and may be contaminated by the germs of scarlatina, typhoid fever and even tuberculosis. Hence the advisability, or rather the necessity, of scalding, sterilizing or boiling.

In regard to the quantity of milk to be given at each feeding, too much care cannot be exercised. The capacity of an infant's stomach five days old is seven and a half drachms; two months, four ounces; twelve months, ten ounces; two years, twenty-five ounces.

An infant is nourished in proportion to his power of digesting the food with which he is supplied, and not in proportion to the quantity of nutritious material he may be induced to swallow. It is an absolute essential to the present and future health of the infant that the digestive organs shall enjoy a due interval of rest between each meal and its successor. Digestion is not completed when the food leaves the stomach, but has to be carried to its conclusion in the duodenum. Moreover, it should be observed that the contents of the stomach increase in acidity progressively during the digestive act, and that to introduce new food into that viscus when it contains old materials at a maximum degree of acidity, is to prejudice the digestion of the former. Every addition of food to a satisfied stomach is only a new labor to a tired digestion. It necessarily follows that the digestive power is overtaxed, unless emesis takes place; the partly digested milk passes into the intestines to produce the usual train of attendant evils.

Since individuals differ in their capacity and physiological demands, a fixed rule cannot be given to regulate the amount of food. The following index is suggested by Dr. Slifer:

| AGE. | INTERVALS. | AMOUNT. |
|---|---|---|
| 1 month | Every 2 hours | 1 ounce. |
| 2 months | during the | 2 ounces. |
| 3 months | day | 3 " |
| 4 months | | |
| 5 months | Every 2½ hours | 4 " |
| 6 months | during the | 5 " |
| 7 months | day | 6 " |
| 8 months | | |
| 9 months | Every 3 hours | 6 " |
| 10 months | during the | to |
| 11 months | day | 10 " |
| 12 months | | |

Feed as little as possible between the hours of 9 p. m. and 5 a. m., thus giving the child ample time for sleep. Some children can do with one feeding, others demand food two or three times during the night. As the child advances in age night feeding should be decreased and the hours of sleep prolonged. Infants under twelve months of age should feed exclusively from either the breast or the bottle.

Food enters the system in two ways: by the stomach and by the lungs. Respiration is necessarily a nutritive function, and the constant waste and repair of the cell elements of the body as much require for their transmutation the oxygen of the air as the hydrocarbons, albuminoids, and fats of the body. As a rule the lungs are not considered as an organ of nutrition. But examine the functions of respiration closely, and there can be no doubt upon that point. Every one knows that without oxygen no one can live even five minutes. Oxygen, though generally classed as a fuel food, as a tissue food has no equal.

Dr. Griscom says: "Food carried from the stomach to the blood cannot become nutritive till it is properly oxygenated in the lungs: so that a small quantity of food, if less wholesome, may be made nutritive by pure air as it passes through the lungs. But the best of food cannot be changed into pure blood till it is vitalized by pure air in the lungs. Sixty-eight hundredths of our body is made up of oxygen. How necessary then is it that babies should have an abundant supply of it through proper ventilation, pure atmosphere, outdoor rides or walks, the effect of which is to stimulate circulation and satisfy the demands of tissue cells for oxygen. This done, there is in infantile life but little left for drugs to accomplish."

I trust I have succeeded in establishing the fact that faulty and excessive alimentation are the main causes of disorders and diseases in the first year of infantile life. The suggestions herein given are gathered from authorities and special study on the subject. If strictly adhered to they will reduce mortality to its minimum. As stated in the beginning, the digestive organs constitute the nidus of disease in the first year. Primarily these disorders are simply functional through the irritation of the nervous system. But if the digestive inactivity be not remedied promptly a true inflammatory enteritis may be the result. We may witness a septic condition produced by the torpidity of the glandular or lymphatic system, and rachitis may follow. Again, too much burden may be thrown upon the liver, whose function is the secretion of bile, the manufacture and storage of sugar, and the destruction of worn-out blood-cells. The liver is much more prominent in infancy than in adult life. When too long overtaxed by indigestion, it can neither dehydrate the material which courses through it, nor maintain its glycogenic functions, nor throw off those refuse matters that are discharged into it. Hence, autoinfection, sepsis, and all its dreadful concomitants.

And now, gentlemen, that we are entering the season of the year during which intestinal disorders among infants give us a mortality that is appalling, if you are interested in the rearing and welfare of an infant, I trust that after you have patiently listened to the above, you may exclaim in the language of Addison, "The Bane and Antidote lie both before me!" I claim neither authorship nor originality. My sole object is: *Save the babies!*

102 *Whitehall St.*

